# Cardiac metastasis mimicking acute myocardial infarction in a patient with urachal carcinoma: a case report and diagnostic dilemma

**DOI:** 10.3389/fcvm.2025.1672655

**Published:** 2025-12-18

**Authors:** Huanglie Xie, Ke Zhang, Pengyu Han, Chaoyang Zheng

**Affiliations:** 1 The Second Affiliated Hospital of Guangzhou University of Chinese Medicine, Guangzhou, China; 2 Second Clinical Medical College, Guangzhou University of Chinese Medicine, Guangzhou, China

**Keywords:** acute myocardial infarction, differential diagnosis, electrocardiogram, pericardial metastasis, urachal carcinoma

## Abstract

Urachal carcinoma (Ur C) is a rare invasive tumor, prevalent in males, with a poor prognosis, and most of them are in an advanced stage at the time of diagnosis. Cardiac metastatic tumors are relatively rare and usually originate from distant metastases of advanced malignant tumors. When the tumor infiltrates the myocardial tissue and affects the repolarization of the diseased myocardium, the electrocardiogram may show changes in the ST-T segment, which is similar to the electrocardiogram of acute myocardial infarction. During clinical diagnosis and treatment, cardiac metastases are mostly easy to be misdiagnosed or overlooked when the relevant tumor history is unknown. In this paper, we discuss a case of cardiac metastasis of urachal carcinoma suspected to be acute myocardial infarction, and analyze and summarize its clinical features, diagnostic and therapeutic process, and abnormal electrocardiographic changes. In order to provide a reference for the diagnosis and treatment of patients with pericardial metastasis of clinically malignant tumors.

## 1 Introduction

Acute myocardial infarction (AMI) is a major global cause of disability and mortality. Recent trends show a younger onset age for AMI, with rising incidence and hospitalization rates (1). Early electrocardiographic (ECG) identification is an important part of the clinical diagnosis and treatment of this disease. Cardiac metastatic tumor is a rare disease with a poor prognosis and an incidence of 0.7%–3.5% (2). It mostly originates from distant metastasis of some advanced malignant tumors. Cardiac metastases of malignant tumors can be difficult to distinguish from ECG changes of acute myocardial infarction. They require sufficient attention in clinical practice.

## 2 Case reports

**Figure 1:**
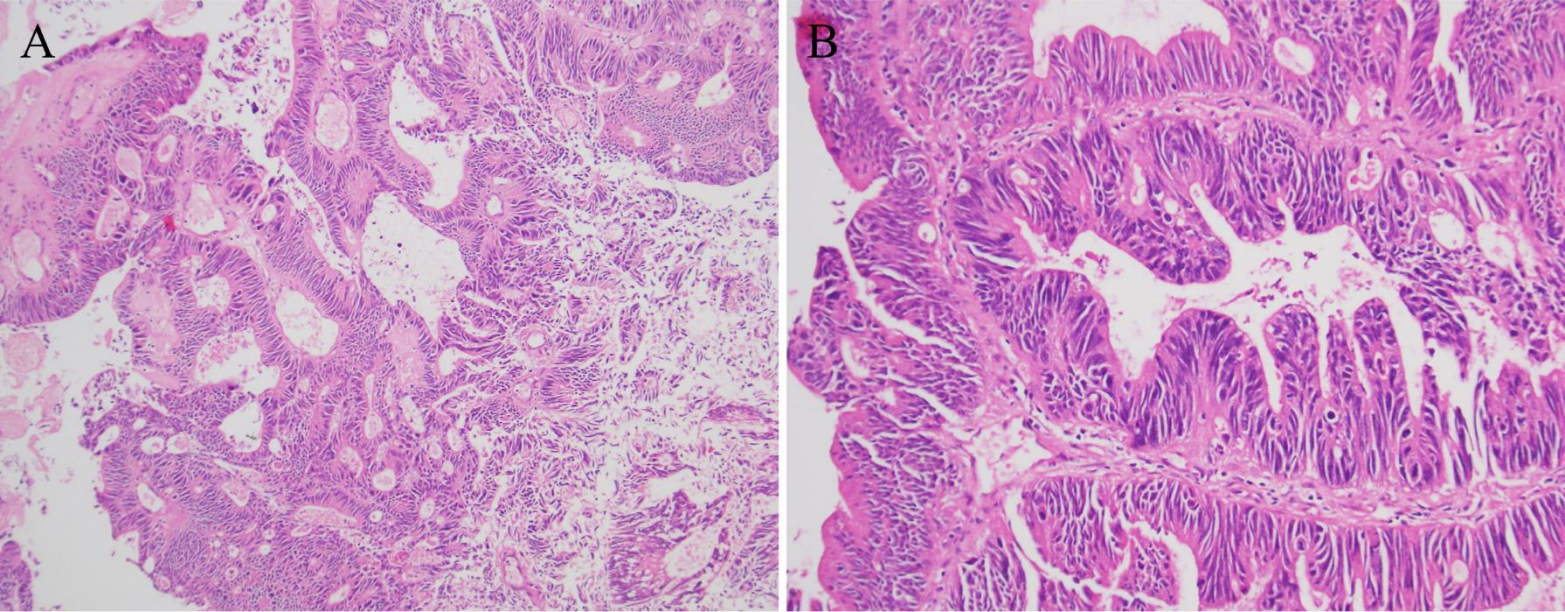
Pathological specimen. Pathological diagnosis: urethral cyst and moderately differentiated bladder adenocarcinoma. Based on immunohistochemical results and clinical history, findings **(A)** and **(B)** are consistent with intestinal-type adenocarcinoma of urethral cyst.

**Figure 2:**
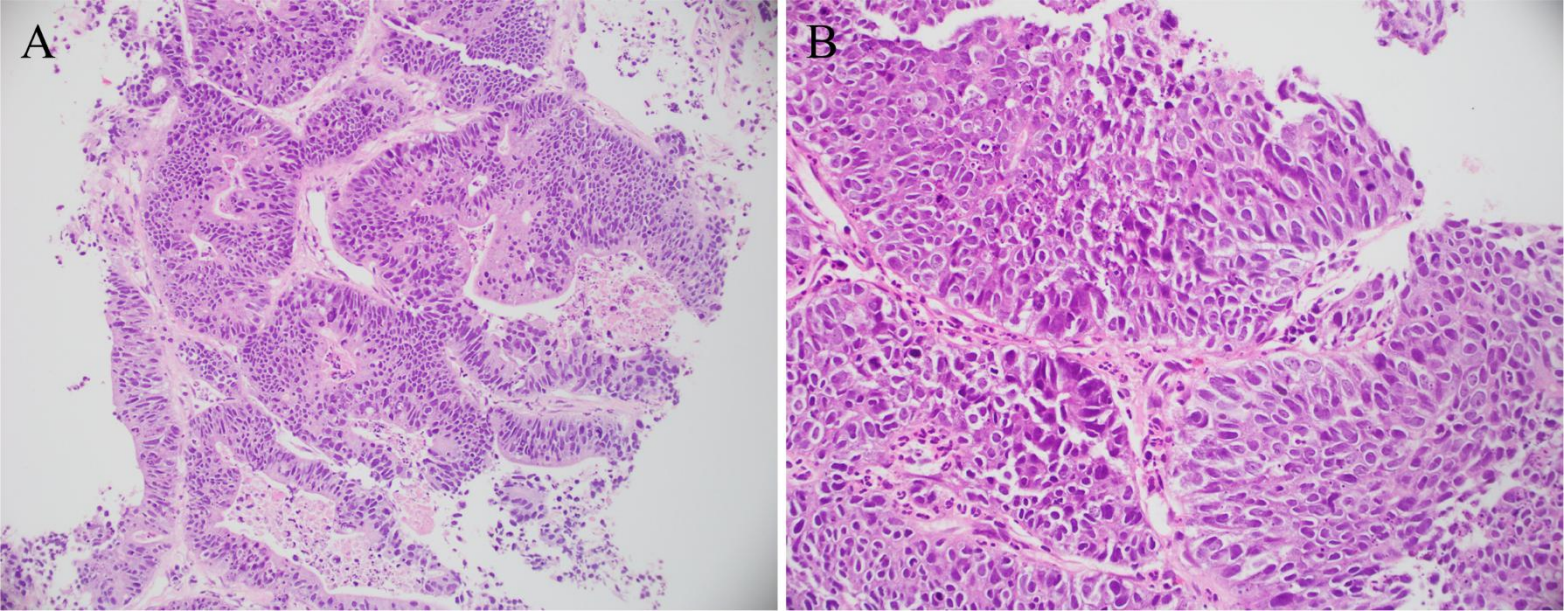
Pathological specimen. Pathological diagnosis: the provided lung tissue shows evidence of adenocarcinoma. In conjunction with immunohistochemical results and medical history, findings **(A)** and **(B)** are consistent with metastatic urethral cystic carcinoma.

Male patient, 60 years old. In 2023, due to hematuria, a positron emission tomography (PET) scan performed at another hospital suggested possible urachal carcinoma with potential pulmonary metastases. In February 2023, he underwent umbilical umbilical urachal lesion resection, and partial cystectomy at our hospital for urachal carcinoma. The postoperative pathology report indicated an adenocarcinoma of the intestinal type (see Figure 1). From March to July of 2023, the patient underwent 8 courses of systemic chemotherapy of erbitux (C225) + leucovorin, fluorouracil, and oxaliplatin (FOLFOX) in our hospital, and the therapeutic effect remained stable. An enhanced CT examination of the chest was performed during the chemotherapy period, and lung metastasis was suspected. In November 2023, adenocarcinoma was found in lung tissue, consistent with the metastasis of urachal carcinoma, as indicated by the patient's history and immunohistochemistry results (see Figure 2). Thereafter, the treatment plan was changed, and 8 courses of bvacizumab (BEV) + leucovorin, fluorouracil, and irinotecan (FOLFIRI) regimen were administered from December 2023 to July 2024. On 26 August, 2024, the patient began to experience chest tightness and pain, lasting 10–30 min each time, with intermittent episodes, mainly in the subxiphoid and precordial regions, unrelated to activities, accompanied by dyspnea, which interfered with sleep, and was slightly relieved by taking heart pills. The patient visited our hospital's outpatient clinic, where troponin levels were found to be 0.084 ug/L (reference value: 0–0.014 ug/L), so the patient was arranged for further treatment in the cardiovascular department. Previous history of diabetes mellitus for 20 years, hyperlipidemia for 1 year, alcohol consumption for 40 years, no smoking history. family or psychosocial history were not reported. Physical examination: T 36.1 ℃, BP 130/88 mmHg, P 80 beats/min, and R 20 beats/min. Conscious and clear-minded. Both lungs were breathing soundly and clearly. There was no obvious bulge in the precordial area. The heart rhythm was regular, and the heart sounds were distant/muffled. No abnormalities were seen during the rest of the physical examination.

**Figure 3:**
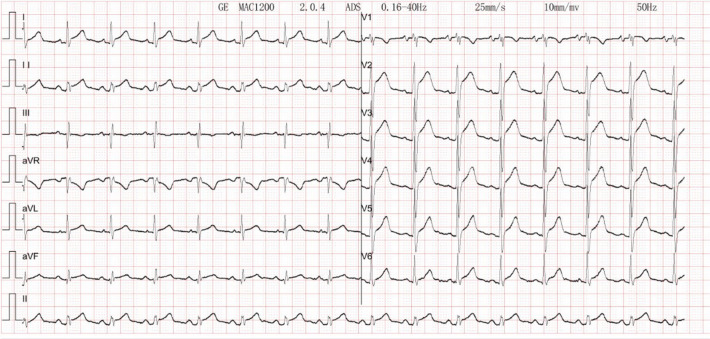
Electrocardiogram. There is sinus rhythm on the ECG. The aVR lead's PR segment is boosted. The ST segments leads I, II, V2, V3, V4, V5, and V6 show concave elevation.

**Figure 4:**
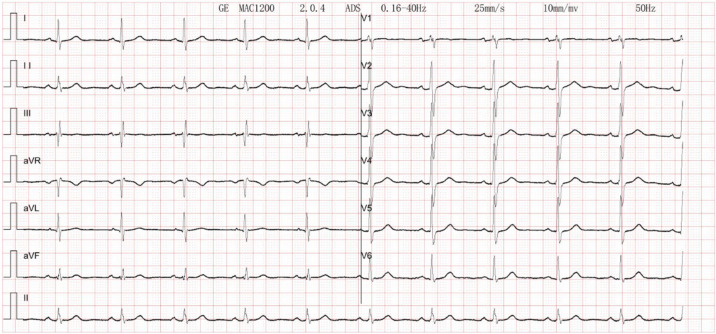
Electrocardiogram. Normal electrocardiogram.

**Figure 5:**
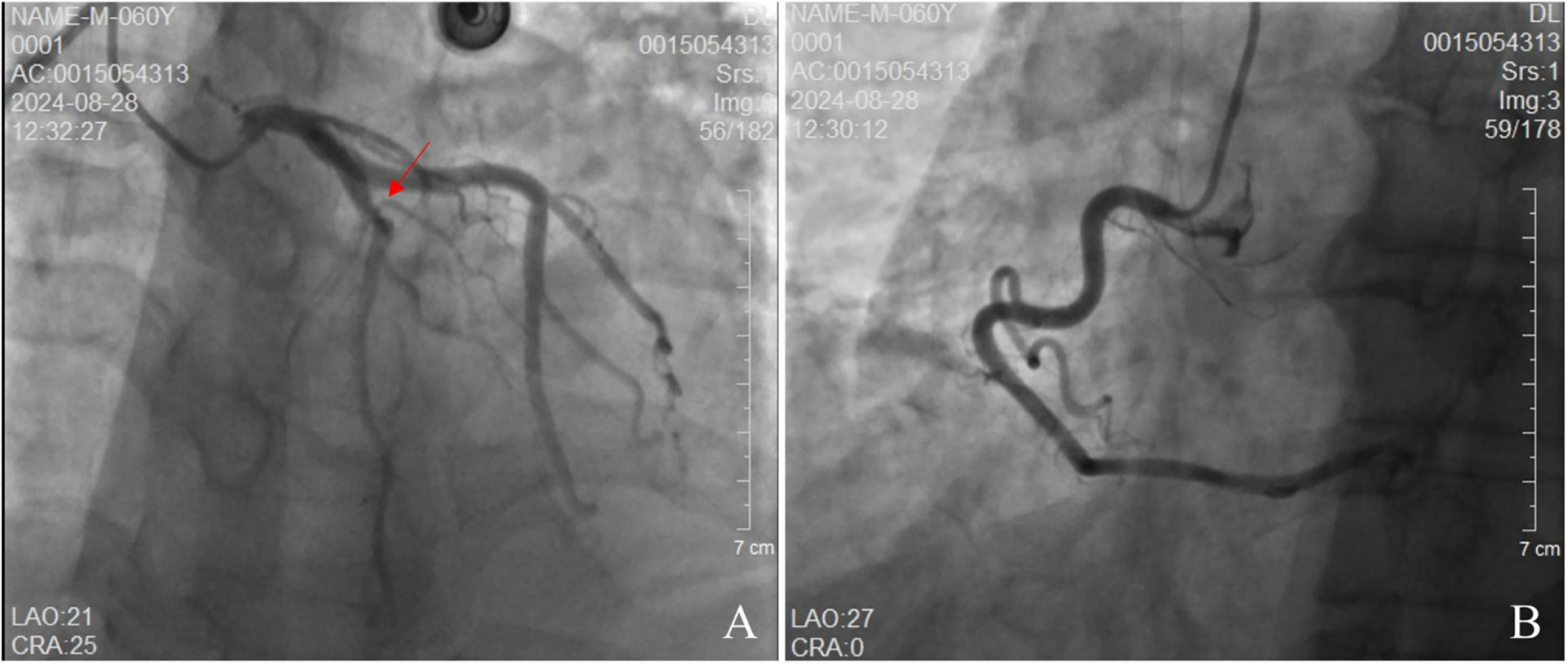
Coronary angiography. **(A)** demonstrates no stenosis in the left main artery. The left anterior descending (LAD) branch exhibits approximately 60% stenosis, while the left circumflex artery shows no stenosis. **(B)** reveals no stenosis in the right circumflex artery.

**Figure 6:**
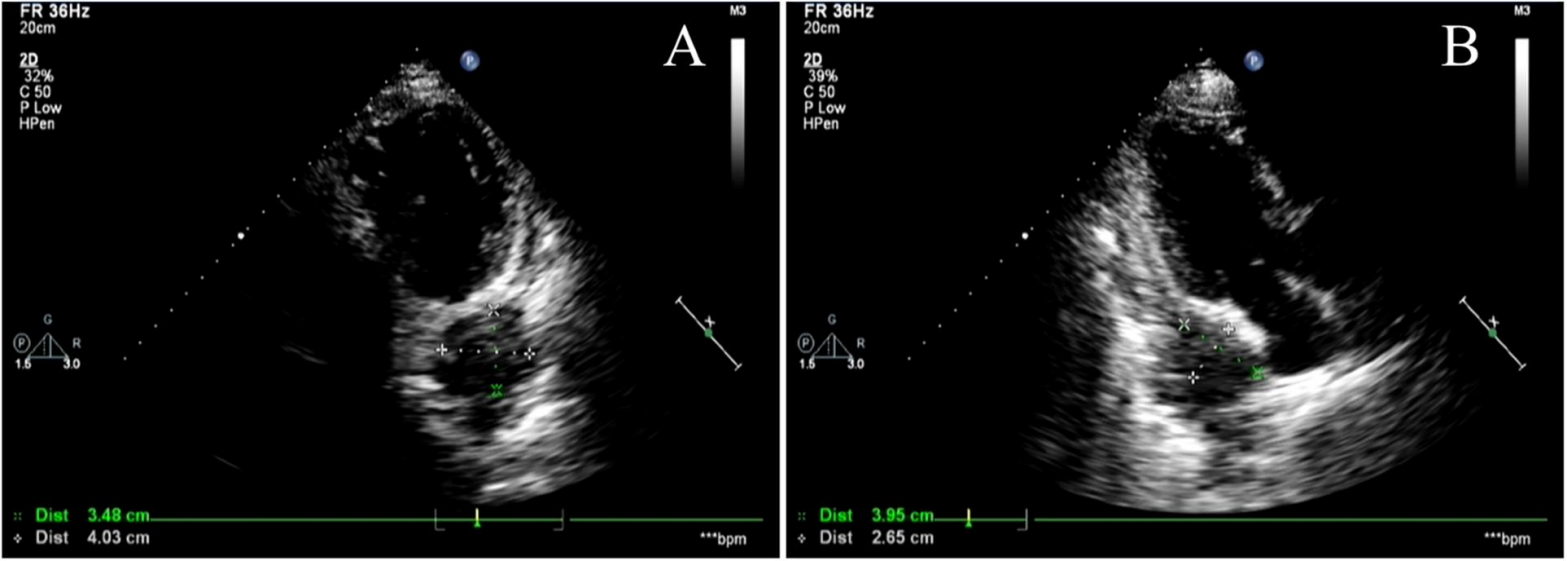
Cardiac Doppler ultrasound. **(A,B)** show different views of cardiac color Doppler ultrasound revealing a localized hyperechoic lesion measuring approximately 40 × 35 mm in the posterolateral region of the left atrium, exhibiting mild hypoechoic characteristics. Metastatic lesions cannot be excluded.

The patient's electrocardiogram was perfected on August 28, 2024 (see Figure 3), and compared with the ECG from July 15, 2024 (see Figure 4), changes in ST-segment elevation were observed in leads I, II, aVL, aVF, and V2–V5. Combined with the patient's symptoms, signs, elevated ultrasensitive troponin, and other indicators, an acute myocardial infarction was diagnosed. A coronary angiography was performed on August 28, 2024. No stenosis was seen in the left main stem (LM), the proximal stenosis in the left anterior descending branch (LAD) was about 60%, and no significant stenosis was seen in the left circumflex branch (LCX) or the right coronary artery (RCA). Coronary angiography results (see Figure 5). Combined with the results of coronary angiography, the diagnosis of acute myocardial infarction could be excluded. Medically, symptomatic treatments such as antiplatelet aggregation and lipid-lowering were given. During hospitalization, a cardiac ultrasound revealed a small amount of pericardial effusion (5 mm at the posterior left ventricular wall and 7 mm at the lateral wall) and limited abnormal echogenicity in the posterior lateral aspect of the left atrium. This area measured approximately 40 by 35 mm, was slightly hypoechoic, and the nature of the echogenicity was undetermined. Metastasis was not excluded based on the patient's history (see Figure 6). The patient continued with the ninth cycle of BEV + FOLFIRI chemotherapy on September 2, 2024, during which they experienced discomfort including coughing and shortness of breath. On October 5, 2024, they were readmitted due to worsening symptoms. The patient's condition deteriorated, and he elected to discontinue active treatment. He subsequently died. The patient did not undergo pulmonary angiography, cardiac tissue biopsy, autopsy, or histopathological diagnosis. The final clinical diagnosis was: malignant tumor of the umbilical urachal carcinoma, intestinal-type adenocarcinoma (stage IV, with metastases to the lungs, adrenal glands, pericardium, and lymph nodes), and secondary malignant tumor of the pericardium.

## 3 Discussion

The patient presented with suspected acute myocardial infarction at the initial visit. The patient presented with chest tightness and pain. Combined with elevated troponin levels and ST-segment elevation on the electrocardiogram, the initial diagnosis was suspected acute myocardial infarction. However, the patient's chest tightness and pain were intermittent and unrelated to physical exertion. The electrocardiogram showed widespread ST-segment depression rather than elevation, and did not indicate a specific location of the infarction. Subsequent coronary angiography ruled out the possibility of acute myocardial infarction. Combining the patient's cardiac echocardiogram, ECG and the cardiac angiography with the patient's tumor history we established cardiac metastases as the most likely working diagnosis. Clinically, the presence of cardiac metastases from tumors is an infrequent occurrence, and their identification is often challenging due to the absence of specific clinical manifestations. The pericardium is the most commonly involved site (3). Along with cardiac involvement, patients may present with symptoms and signs such as chest tightness and chest pain, shortness of breath, dyspnea, tachycardia, and pericardial friction sounds. Cardiac metastases do not have specific electrocardiographic manifestations, and the most common electrocardiographic changes are nonspecific ST-segment and T-wave abnormalities, which may result from local myocardial injury or ischemia caused by the metastatic tumor.

The patient in this case had an advanced malignant tumor with a poor prognosis. The patient's cardiac ultrasound and electrocardiogram exhibited abnormal changes at the time of admission, which, when combined with the clinical manifestations of dyspnea and chest pain, as well as the history of a tumor, were indicative of acute pericarditis, a condition believed to be caused by the tumor's invasion of the heart. Clinically, acute myocardial infarction, pericarditis, myocarditis, and pulmonary causes such as pulmonary embolism require differential diagnosis. A common ECG change in pericarditis is diffuse PR segment depression with concave elevation of ST segments in other leads. Pericardial effusion is frequently visible on cardiac echocardiography. The ECG of typical ST-segment elevation myocardial infarction may show “tombstone-like” ST-segment changes, pathological Q waves, and often localizes the infarct site. Patients with pulmonary embolism often present with dyspnea as the primary symptom. Electrocardiograms commonly show sinus tachycardia, and some patients exhibit the “SIQ-III-TIII sign.” Chest tightness or pain caused by acute myocardial infarction often worsens with exertion or physical activity, whereas chest tightness or pain resulting from pericarditis is unrelated to activity. Myocarditis is most commonly seen in young and middle-aged adults, and is generally preceded by symptoms such as fever, cough, and diarrhea. Although electrocardiography is a crucial clinical tool for distinguishing pericarditis from myocardial infarction, the two conditions may coexist, often complicating clinical diagnosis (4, 5). The patient's electrocardiogram showed concave elevation of ST segments in I, II, aVL, aVF, and V2–V5. It was considered that the tumor infiltration of cardiac tissue affected the repolarization of the diseased myocardium, resulting in ST-segment changes in the electrocardiogram, and the mechanism of which may be related to the presence of damage currents at the site of the lesion (6).

Urachal carcinoma was first documented by Hue and Jacquin in 1863. It remains a rare clinical malignancy of the urinary system, with an annual incidence of approximately one in a million, accounting for 0.35%–0.7% of bladder tumors (7). Among these, adenocarcinoma is the most prevalent type of urachal carcinoma, comprising 10%–30% of adenocarcinomas of the bladder (8, 9). In this case, the patient exhibited a case of umbilical urachal cancer that had metastasized to the lungs and subsequently to the heart. The cardiac metastatic tumor was duly considered. The incidence of cardiac metastatic tumors in autopsies of cancer patients has been reported to be as high as 1.5%–25% (10, 11). Clinically, the heart is often neglected as a target organ for tumors, resulting in a relative paucity of research on cardiac metastatic tumors. The diagnosis of cardiac tumors often relies on the use of multiple imaging techniques, including cardiac computed tomography (CT), cardiovascular magnetic resonance imaging (CMR), and echocardiography (12) the use of color Tissue Doppler Imaging (TDI) and pulsed-wave TDI could have facilitated a more accurate diagnostic interpretation of the suspected intracardiac mass. They hold diagnostic and prognostic value in identifying intracardiac masses and distinguishing between tumors, vegetations, and thrombi (13, 14).

A clinical study of the electrocardiographic features of cardiac metastatic tumors showed that ST-segment elevation in these patients exhibited the following characteristics: absence of pathologic Q and R-wave deletions, a negative terminal T-wave in the ST-segment elevation leads, and no electrocardiographic evolution (4). Clinically, the presence of ST-segment elevation on the ECG is mostly considered a characteristic change of acute myocardial infarction, except in some patients. It is highly susceptible to misdiagnosis if not combined with other clinical data. In the context of cardiac tumors, myocardial infarction-like changes on electrocardiography (ECG) are a prevalent manifestation. However, the precise identification of these changes is paramount for the timely diagnosis and effective treatment of patients. With this case, we emphasize that “red flags” in a patient with a known cancer history that should prompt consideration of cardiac metastasis, even in the presence of a STEMI-mimicking ECG.

This study has the following limitations: The most significant limitation is the absence of histopathological confirmation of the cardiac metastasis;As a case report, the findings are not generalizable. The clinical courses and manifestations of other patients may vary greatly; The report is descriptive and retrospective, limiting the ability to establish causality or draw broader conclusions about management strategies.

## Data Availability

The original contributions presented in the study are included in the article/Supplementary Material, further inquiries can be directed to the corresponding author.
