# Correction: Cardiac metastasis mimicking acute myocardial infarction in a patient with urachal carcinoma: a case report and diagnostic dilemma

**DOI:** 10.3389/fcvm.2026.1776023

**Published:** 2026-02-26

**Authors:** Huanglie Xie, Ke Zhang, Pengyu Han, Chaoyang Zheng

**Affiliations:** 1 The Second Affiliated Hospital of Guangzhou University of Chinese Medicine, Guangzhou, China; 2 Second Clinical Medical College, Guangzhou University of Chinese Medicine, Guangzhou, China

**Keywords:** acute myocardial infarction, differential diagnosis, electrocardiogram, pericardial metastasis, urachal carcinoma

An incorrect number was provided for the Guangdong Provincial Hospital of Traditional Chinese Medicine's Special Research Fund for Traditional Chinese Medicine Science and Technology. The correct numbers are YN2022QN28 and YN2022MS08.

The footnote “^1^C225: Cetuximab; FOLFOX regimen: Folinic acid (FOL) + Fluorouracil (F) + Oxaliplatin (OX); BEV: Bevacizumab; FOLFIRI regimen: Folinic acid (FOL) + Fluorouracil (F) + Irinotecan (IRI)” has been removed.

Author Pengyu Han was erroneously spelled as Pengyu Hang.

The original version of this article has been updated.

