# Supplementary figures and images for: Cardiac metastasis mimicking acute myocardial infarction in a patient with urachal carcinoma: a case report and diagnostic dilemma
Source: Front Cardiovasc Med. 2025 Dec 18;12:1672655. doi: 10.3389/fcvm.2025.1672655 (PMC12756384; doi:10.3389/fcvm.2025.1672655)

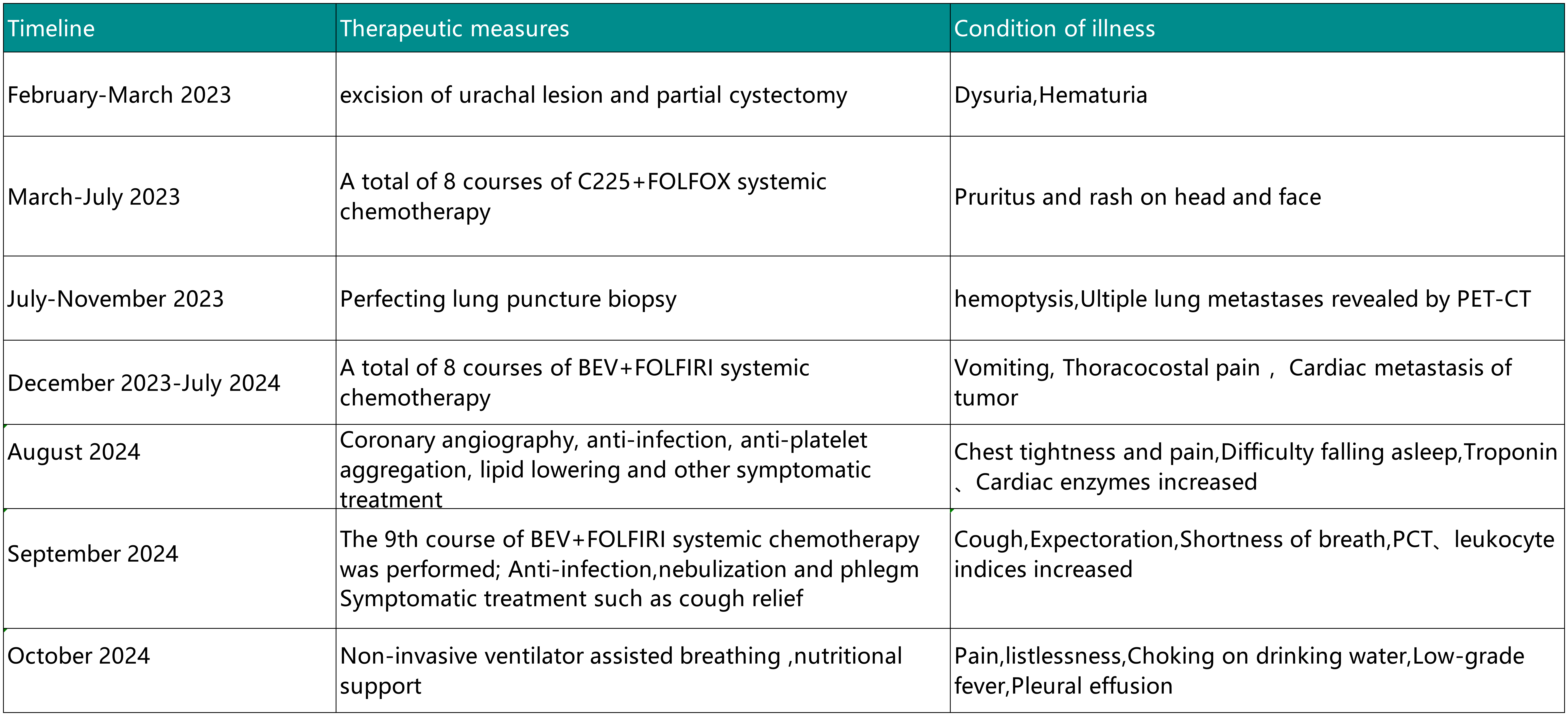

Supplement: Supplementary file 1 [file Image1.tif]
